# Renal arterial branch calcification mimicking renal calculi in ultrasonography

**DOI:** 10.4103/0971-4065.50681

**Published:** 2009-01

**Authors:** B. Vijayalakshmidevi, S. Sarala, A. Y. Lakshmi, G. Sivaramakrishna, V. Sivakumar

**Affiliations:** Department of Radiodiagnosis, Sri Venkateswara Institute of Medical Sciences, Tirupati, India; 1 Department of Nephrology, Sri Venkateswara Institute of Medical Sciences, Tirupati, India

The calcifications in the renal sinus are often due to renal calculi and occasionally due to gas. Calcifications of the branches of the renal artery coursing through the sinus may be mistaken for renal calculi in some rare instances, resulting in a misdiagnosis.

All hyperechoic or echogenic foci in the renal medulla are not caused by stones. Renal stones and gas are the commonly reported echogenic foci in the sinus region. However, in rare cases, the calcified vessel walls of renal arterial branches coursing through the sinus and renal parenchyma may be mistaken for renal calculi. We report herein one such observation.

**Figure 1:**
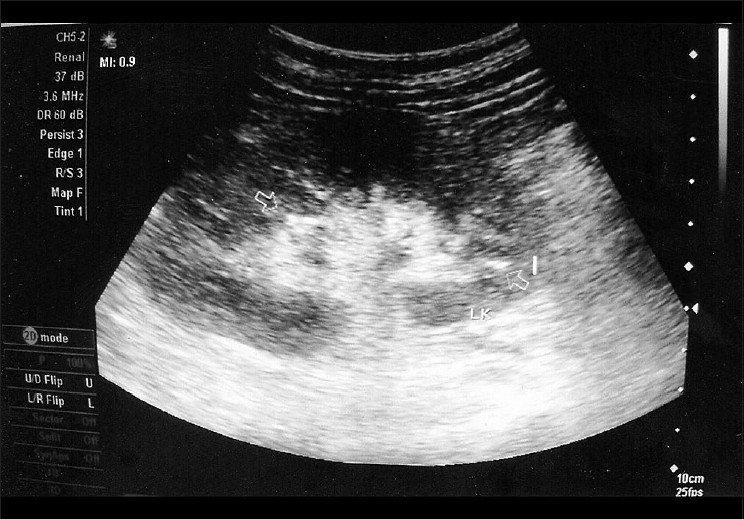
Ultrasound image of the left kidney showing echogenic foci in the renal sinus region (marked with open arrow)

**Figure 2:**
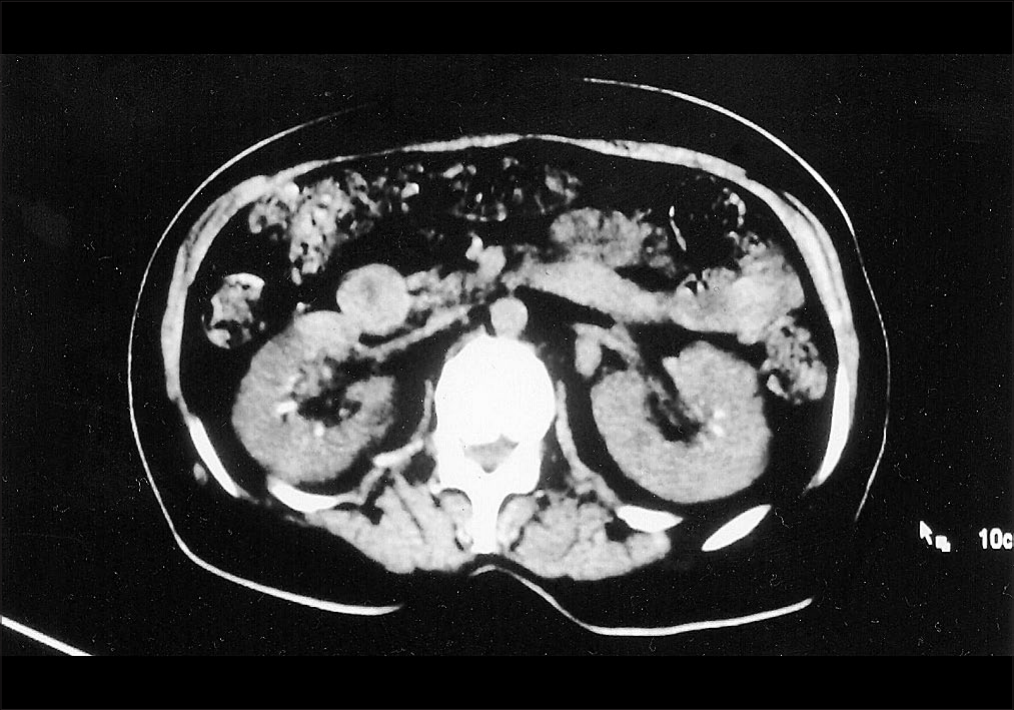
Unenhanced computed tomography showing multiple calcific densities in the segmental renal artery and in its branches on both sides

A middle-aged woman diagnosed with diabetic nephropathy and hypertension presented with symptoms of urinary tract infection. Ultrasound of the abdomen revealed multiple calcifications at the junctional region of the sinus echoes and the medulla in both the kidneys [Figure 1]. Although the calcifications were initially interpreted as renal stones, in view of their unconventional anatomical location, the patient was further subjected to Doppler ultrasonography and plain computed tomography which revealed the presence of calcification in the segmental and interlobar arteries, which was mistaken to be stones [Figure 2]. During their course through the sinomedullary region, calcified branches of the renal artery, such as the segmental, interlobar, and arcuate arteries, may mimic nephrolithiasis[1] in ultrasonographic imaging. In such a situation, a Doppler study is helpful in demonstrating the actual origin of calcification from the vessel wall to be distinct from the curvilinear appearance of calculi.[2] A noncontrast CT study will also help to confirm the exact origin of calcification when it is observed in renal artery branches.

We present here one such observation wherein an initial misdiagnosis of renal stone disease was later corrected to be renal calcification secondary to vascular calcification.
